# Enduring Mental Health Morbidity and Social Function Impairment in World Trade Center Rescue, Recovery, and Cleanup Workers: The Psychological Dimension of an Environmental Health Disaster

**DOI:** 10.1289/ehp.11164

**Published:** 2008-05-13

**Authors:** Jeanne Mager Stellman, Rebecca P. Smith, Craig L. Katz, Vansh Sharma, Dennis S. Charney, Robin Herbert, Jacqueline Moline, Benjamin J. Luft, Steven Markowitz, Iris Udasin, Denise Harrison, Sherry Baron, Philip J. Landrigan, Stephen M. Levin, Steven Southwick

**Affiliations:** 1 Department of Psychiatry, Mount Sinai School of Medicine, New York, New York, USA; 2 Department of Environmental and Occupational Health, School of Public Health, SUNY-Downstate, Brooklyn, New York, USA; 3 Department of Community and Preventive Medicine, Mount Sinai School of Medicine, New York, New York, USA; 4 Department of Medicine, SUNY-Stony Brook, Stony Brook, New York, USA; 5 Queens College, City University of New York, Flushing, New York, USA; 6 University of Medicine and Dentistry of New Jersey, Piscataway, New Jersey, USA; 7 Bellevue Hospital Center/New York University School of Medicine, New York, New York, USA; 8 Division of Surveillance, Hazard Evaluations, and Field Studies, National Institute for Occupational Safety and Health, Centers for Disease Control, Washington, DC, USA; 9 Department of Psychiatry, Yale Medical School, VA Connecticut Healthcare System, National Center for Post-traumatic Stress Disorder, West Haven, Connecticut, USA

**Keywords:** depression, disaster workers, functional impairment, occupational health, post-traumatic stress disorder, stress, World Trade Center

## Abstract

**Background:**

The World Trade Center (WTC) attacks exposed thousands of workers to hazardous environmental conditions and psychological trauma. In 2002, to assess the health of these workers, Congress directed the National Institute for Occupational Safety and Health to establish the WTC Medical Monitoring and Treatment Program. This program has established a large cohort of WTC rescue, recovery, and cleanup workers. We previously documented extensive pulmonary dysfunction in this cohort related to toxic environmental exposures.

**Objectives:**

Our objective in this study was to describe mental health outcomes, social function impairment, and psychiatric comorbidity in the WTC worker cohort, as well as perceived symptomatology in workers’ children.

**Methods:**

Ten to 61 months after the WTC attack, 10,132 WTC workers completed a self-administered mental health questionnaire.

**Results:**

Of the workers who completd the questionnaire, 11.1% met criteria for probable post-traumatic stress disorder (PTSD), 8.8% met criteria for probable depression, 5.0% met criteria for probable panic disorder, and 62% met criteria for substantial stress reaction. PTSD prevalence was comparable to that seen in returning Afghanistan war veterans and was much higher than in the U.S. general population. Point prevalence declined from 13.5% to 9.7% over the 5 years of observation. Comorbidity was extensive and included extremely high risks for impairment of social function. PTSD was significantly associated with loss of family members and friends, disruption of family, work, and social life, and higher rates of behavioral symptoms in children of workers.

**Conclusions:**

Working in 9/11 recovery operations is associated with chronic impairment of mental health and social functioning. Psychological distress and psychopathology in WTC workers greatly exceed population norms. Surveillance and treatment programs continue to be needed.

It is estimated that between 40,000 and 92,000 men and women were involved in the rescue, recovery, and cleanup operations that followed the 11 September 2001 (9/11), terrorist attacks on the World Trade Center (WTC), depending on the criteria used for cohort eligibility [Centers for Disease Control and Prevention (CDC) 2004a, 2004b; Murphy et al. 2007]. Service in these operations exposed workers to toxic and unsafe working conditions, including smoke, fumes, and highly alkaline dust (pH 10–11). We previously reported on elevated rates of pulmonary symptomatology in a cohort of WTC workers (Landrigan et al. 2004). In addition to hazardous chemical and physical environmental exposures, the working conditions at the WTC involved exposures to serious psychosocial stressors, including long hours and arduous work, treacherous and chaotic working conditions, fear for personal safety, and handling body parts and personal effects of victims, or working in close proximity to such operations (Landrigan et al. 2004; Neria et al. 2006; Platner 2002).

Psychological stress and trauma, particularly when chronic, can interact with chemical and physical environmental exposures in ways that are still not well understood and can exacerbate or contribute to the development of a wide range of medical conditions, including cardiovascular, pulmonary, gastrointestinal, neurologic, and autoimmune disorders (McEwen and Lasley 2002). There is evidence that stressful conditions can increase susceptibility to toxic insult (Peters et al. 2007), and environmental researchers are considering the various ways in which mental health fits into the conceptual framework of environmental health sciences (Schmidt 2007).

Psychological stress and trauma can also cause or exacerbate psychiatric disorders. The best known of the psychiatric responses to stress is posttraumatic stress disorder (PTSD). PTSD is characterized by repetitive reexperiencing of the traumatic event in the form of intrusive and unwanted memories of the trauma; nightmares and flashbacks about the trauma; difficulty modulating arousal as evidenced by insomnia, irritability, angry outbursts, hypervigilance, difficulty concentrating, and exaggerated startle response; avoidance of stimuli associated with the trauma; and a general numbing of emotions with a feeling of detachment from others. Elevated rates of PTSD have previously been reported in a number of studies of WTC rescue and recovery workers (CDC 2004b; Difede et al. 1997; Gross et al. 2006; Perrin et al. 2007; Tapp et al. 2005).

Other psychological disorders have also been associated with traumatic stress, including depression and panic disorder (Kessler et al. 2005). Major depressive disorder is defined as depressed mood or loss of interest or pleasure in nearly all activities for a period of at least 2 weeks [American Psychiatric Association (APA) 2000]. Other symptoms include significant weight gain or weight loss when not dieting, insomnia or hypersomnia, slowed and retarded movement or hyperactive movement, fatigue or loss of energy, excess or inappropriate guilt or feelings of worthlessness, indecisiveness or difficulty thinking and concentrating, and recurrent thoughts of death. Panic disorder is characterized by recurrent, unexpected panic attacks marked by discrete periods of intense fear or discomfort accompanied by a least four of the following symptoms: palpitations; pounding heart or accelerated heart rate; sweating, trembling, or shaking; sensations of shortness of breath or smothering; feeling of choking; chest pain or discomfort; nausea or abdominal distress; feeling dizzy, unsteady, lightheaded, or faint; feelings of unreality or detachment; fear of losing control or going crazy; fear of dying; numbness or tingling; and chills or hot flashes (APA 2000).

PTSD, depression, and panic disorder associated with traumatic stress often co-occur in the same individual, a situation referred to as comorbidity. Rates of psychiatric comorbidity have been found to be high in community, at-risk, and clinical populations of individuals who have been diagnosed with PTSD (Resick 2001). For example, in the National Comorbidity Study, a community sample assessing the rates of mental disorders in the general population, Kessler et al. (2005) reported that among subjects diagnosed with PTSD, 88% of men and 79% of women also met criteria for at least one other comorbid mental disorder.

In the present analysis, as part of the WTC Medical Monitoring and Treatment Program (MMTP) supported by the National Institute for Occupational Safety and Health (NIOSH), we expanded our earlier report of 1,138 WTC workers in which we described prevalence rates of probable PTSD, major depression, and panic disorder. In a much larger sample of this cohort, we investigated prevalence of probable PTSD over a 5-year period and the prevalence of major depression and panic disorder (CDC 2004b). In addition, we examined psychiatric comorbidity as well as on the extensive symptomatology detected in workers who do not meet all of the diagnostic criteria for one of these psychiatric disorders. We also assessed the degree to which probable psychiatric disorders, comorbid psychiatric disorders, and substantial stress reactions are related to impairment in functioning, as measured by problems with alcohol and disruption of social functioning at work and with friends and family. Finally, we examined workers’ beliefs about behavioral symptoms in their children. Overall, these data provide a more complete understanding of the enduring psychiatric burden experienced by this cohort of WTC workers.

## Methods

In 2002 Congress directed the CDC to establish a regional clinical consortium to provide medical and mental health monitoring of WTC rescue and recovery workers. In response, the WTC–MMTP was established, with the Department of Community and Preventive Medicine, Mount Sinai School of Medicine, as the coordinating entity and the Bellevue/New York University Occupational and Environmental Medicine Clinic, the SUNY (State University of New York)-Stony Brook/Long Island Occupational and Environmental Health Center, the Center for the Biology of Natural Systems at Queens College in New York, and the Clinical Center of the Environmental & Occupational Health Sciences Institute at University of Medicine and Dentistry of New Jersey-Robert Wood Johnson Medical School in New Jersey as the other members of the consortium.

Subjects were recruited for participation in a clinical monitoring program through outreach that included union meetings, mailings, media articles, and some 50,000 telephone calls in multiple languages. The first 10,132 participants who completed a self-administered mental health questionnaire and provided written informed consent to permit aggregation of their data into a research database are included in these analyses. Institutional review board approval was obtained at each of the participating institutions. Eligibility for the clinical examination required either having worked or volunteered as part of the rescue, recovery, restoration, or cleanup in Manhattan south of Canal Street, barge-loading piers in Manhattan, or the Staten Island landfill, for at least 24 hr during 11–30 September 2001, or for > 80 hr between 11 September and 31 December 2001. Employees of the Office of the Chief Medical Examiner had no minimum hour requirements. New York City firefighters participated in a separate program.

The clinical examination included a mental health screening questionnaire that used standard instruments to assess emotional status. These included the PTSD Symptom Checklist (PCL; Blanchard et al. 1996); the Patient Health Questionnaire (PHQ) for assessing depression, anxiety, and panic (Spitzer et al. 1999); the CAGE questionnaire for alcohol abuse (Ewing 1984); and the Sheehan Disability Scale to estimate the extent to which emotional problems disrupted work, social life, and family and home responsibilities (Leon et al. 1997). Single items to measure changes in alcohol consumption were also included. Threshold criteria were defined for each measure. Those who met criteria for any scale were referred, on the same day, for clinical evaluation by mental health professionals. These criteria are described in an earlier report on the mental health of the first 1,138 responders examined in the program (CDC 2004b). All workers also underwent physical examinations and had medical and exposure interviews. The questionnaire included a child symptom checklist from the disaster supplement of the Diagnostic Interview Schedule that asked the responder to state whether his or her children exhibited any of 12 symptoms for the period during the responders work on the WTC site and in the month before the examination (Robins 1983).

**Figure 1:**
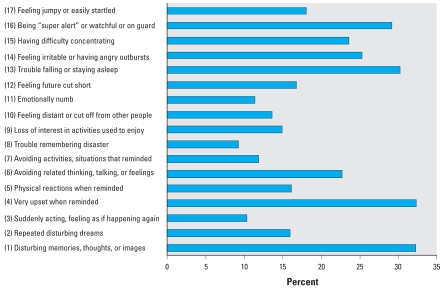
Percentage of responders without probable PTSD diagnosis with positive scores on individual PCL symptom checklist items in the month before examination. Items are taken from the PCL (Blanchard et al. 1996). A response at one of the three highest levels (moderately, quite a bit, or extremely) to a PCL item was considered positive. Numbers in parentheses indicate the item numbers in the checklist.

We calculated three commonly used algorithms for defining probable PTSD. The first two are based on summing up the responses to the 17 Likert-like items in the PCL checklist shown in Figure 1. We classified a responder as having probable PTSD if the score was ≥ 44 or ≥ 50, where each item was scored as 1–5 (corresponding respectively to not at all, a little bit, moderately, quite a bit, or extremely). We also calculated a score using the algorithm from the *Diagnostic and Statistical Manual of Mental Disorders* (APA 2000) as follows. For any item in the checklist, a response at one of the three highest levels (moderately, quite a bit, or extremely) was considered positive. Items were divided into three separate clusters that were scored as follows: cluster B (intrusion)—at least one positive item from items 1–5; cluster C (avoidance)—at least three items from items 6–12; cluster D (hyperarousal)—at least two items from items 13–17. The prevalence of probable PTSD was 20.1%, 11.1%, and 17.1%, respectively, based on these three formulations. We thus used the PCL checklist with a cutoff score of 50 for our definition of probable PTSD in the analyses that follow, as this represented the most conservative estimate for the group as a whole.

Because we are interested in understanding the extent to which mental health symptoms may represent an ongoing problem in members of our population who do not meet diagnostic criteria for probable PTSD, we calculated the frequency distribution of responses to the individual items on the PCL in workers without probable PTSD (responses from moderately to extremely). For comparison purposes, we also tabulated the number of workers who met Schuster’s definition of substantial stress from the post-9/11 national telephone survey (at least one moderately to extremely response to PCL items 1, 4, 13, 14, or 15) (Schuster et al. 2001).

We used PHQ responses and the coding algorithm provided by Spitzer et al. (1999) to assess major depression and panic disorder. A positive response to one of the four CAGE items defined a probable alcohol problem. We defined the interval to first visit as time elapsed between the first day working at the WTC site and date of the first mental health screening examination.

### Statistical analysis

Two-tailed chi-square tests were used to identify associations between probable PTSD and diagnoses for probable major depression or panic disorder with demographic and exposure characteristics of the population and for the reports of child symptomatology. Mantel-Haenszel common odds ratios (ORs) were calculated to provide risk estimates and confidence intervals for PTSD and comorbid mental health outcomes, probable alcohol problem, and reported deaths or injuries in family members or friends as a result of the attacks. We used linear or logistic regression analyses to determine the extent to which the independent variables in Table 1 and comorbid depression predicted the PCL checklist scores or dichotomous outcomes indicating presence or absence of probable PTSD, major depression, alcohol problems, or social disability as measured by the Sheehan scale.

## Results

Table 1 presents demographic characteristics, distribution of hours and days spent in WTC-related activities, and presence at the site during the first 48 hr by our cohort. More than 62% arrived within the first 48 hr after the attack; 84% were present during the first week, and 91% arrived by 24 September 2001. In addition, the majority continued recovery work for ≥ 3 months and thus was present for the early postattack days and also for the arduous and stressful working conditions that followed. Average age of the responders was 42.1 ± 9.1 years (mean ± SD). Associations of probable PTSD, probable major depression, and panic disorder are also shown in Table 1 for each of the respondent characteristics. Significant relationships were observed for probable PTSD with all respondent characteristics except sex; 11.1% met criteria for probable PTSD, 8.8% met criteria for depression, and 5.0% met criteria for panic disorder. We observed widespread symptomatology in the group that was not assigned probable PTSD, panic disorder, or major depression (Figure 1); nearly half (45%) met Schuster’s definition of a substantial stress reaction.

Fewer than 5% of the cohort reported losing members of their family to the attacks, but > 36% reported losing friends. About one-third lost more than one person. We observed significantly elevated rates of probable PTSD [OR = 1.66; 95% confidence interval (CI), 1.21–2.28] and emotional disability (OR = 1.48; 95% CI, 1.16–1.87) associated with having lost a family member to the attack and where disability was measured by the Sheehan scale and signifies emotional disruption of the ability to work or engage in family or social activities. Reported loss of friends resulted in a significant but smaller elevated risk for probable PTSD only (OR =1.2; 95% CI, 1.02–1.43).

More than 17% of the cohort was classified with a probable alcohol problem based on the CAGE. About 24% of alcohol users reported drinking more than usual following 9/11, and about 47% reported that they drank more during the time at which they were working at the WTC in rescue and recovery operations. About one-third reported still drinking more than usual within the month before their clinic visit.

In Table 2, we present data on psychological comorbidity (which we define as the presence of more than one psychiatric condition) and its relationship to emotional disability (Sheehan scale) and alcohol problems. Approximately half of those classified as having probable PTSD also were classified with either probable panic disorder, depression, or both. WTC-responders with probable PTSD had highly elevated ORs for probable depression (OR = 13.9; 95% CI, 11.9–16.2) and panic disorder (OR = 9.2; 95% CI, 7.6–11.1). This comorbidity is associated with being 40–86 times as likely to be rated as disabled, as measured by the Sheehan scale. For the group as a whole, regardless of comorbidity status (Table 2), probable PTSD was associated with more than double the risk for an alcohol problem (OR = 2.3; 95% CI, 2.0–2.5) and more than 17-fold risk for reported social disability (OR = 17.3; 95% CI, 15.1–19.8) compared to those with no psychological morbidity. The point-prevalence of probable PTSD declined from 13.4% at 10 months postattack to 9.3% at 60 months, a rate far higher than in the general population.

Table 3 presents the extent of perceived child symptomatology reported by the workers and by whether or not they have been classified with probable PTSD. The first set of ORs compares the perceptions of those with and without probable PTSD for their children’s symptoms during the time in which the responders were working at the WTC site. The second set of ORs is for the symptoms that these workers perceived their children to be exhibiting in the month before the clinic visit. In all cases the ORs are significantly elevated for workers with PTSD compared to those without the probable diagnosis.

## Discussion

This study documents that the rescue and recovery and cleanup efforts carried out by the workers at the World Trade Center are associated with substantial chronic psychological morbidity and extensive impairment of social functioning. Of 10,132 WTC workers whom we examined, 11.1% had probable PTSD within the month before their mental health examination.

These findings on the high prevalence of PTSD in WTC workers are similar to those encountered in U.S. war veterans. Hoge et al. (2004), using the same PTSD diagnostic checklist that we used and the same cutoff score of 50 on this PCL checklist, found a prevalence of 11.5% for probable PTSD among soldiers returning from Afghanistan. Although not directly comparable to our 1-month prevalence rates, the 12-month prevalence estimates for PTSD among the general adult population in the United States range between 3% and 4% (Kessler et al. 2005).

Consistent with previous studies, PTSD was not the only type of postdisaster psychopathology observed in this cohort. Almost 9% of the cohort met criteria for probable depression during the month in which they were examined, and 5.0% fulfilled diagnostic criteria for probable panic disorder. Another 17% had probable excess use of alcohol. Personal loss of family and friends appears to have increased these rates. Rates of psychological comorbidity were also high. Among the responders with probable PTSD, 12.7% also met criteria for panic disorder or depression, and 1.7% met criteria for all three disorders: probable PTSD, depression, and panic disorder. Of note, approximately half of the workers with probable PTSD also had a probable comorbid psychiatric condition, and these workers were at far higher risk for social dysfunction and alcohol problems. Further, the point prevalence of PTSD in the comorbid group did not decline over time, suggesting that PTSD in this group may be more chronic.

Our results are consistent with other reports in which PTSD has been strongly associated with functional impairment, including interference with occupation, family, school, and leisure activities, among subjects in community samples (Kessler 2000; North et al. 2002), male and female veterans (Kulka et al. 1988; Stein et al. 1997), primary care patients (Zatzick et al. 1997), and female survivors of interpersonal violence (Rapaport et al. 2002). Level of functional impairment associated with PTSD has been comparable to levels observed in severe chronic depression (Laffaye et al. 2003). In our population, the odds ratio for social impairment was significantly elevated more than 17-fold among those with PTSD compared to those without probable PTSD.

Many workers who did not meet study criteria for probable PTSD nevertheless reported suffering from PTSD-related symptoms of stress in the month before their evaluation (Figure 1). For example, approximately one-third of the responders without probable PTSD reported disturbing memories, thoughts, or images; having trouble falling asleep or staying asleep, and being “super-alert.” Nearly half (45%) of all responders without probable PTSD reported suffering from a substantial stress reaction as long as 5 years after the WTC disaster, a rate comparable to the nationally representative sample of U.S. adults surveyed only 3–5 days after the attacks when symptoms typically are at their highest level (Silver et al. 2002).

Most research studies and clinical interventions focus on patients who meet full criteria for PTSD. Our data show that such an approach would fail to meet the needs of many of the responders in the present population who were not classified as having probable PTSD but did have a high prevalence of distressing symptoms and functional impairment. In fact, in the current sample, the likelihood of experiencing marked functional impairment in workers with substantial stress symptoms in the absence of probable PTSD was nearly as elevated as this likelihood for workers with panic disorder alone (OR = 3.3; 95% CI, 2.7–4.0). Focusing attention only on probable psychiatric disorders markedly underestimates the full psychological burden and its social ramifications. It is likely that some of these individuals would benefit from appropriate treatment.

Most WTC workers reported one or more psychological/behavioral symptoms in their children during the time that they worked at the disaster site (Duarte et al. 2006). Stuber et al. (2005) and Schlenger et al. (2002) reported similar findings of substantial WTC-related stress among children in New York City at the time of the disaster and for months afterward. Also consistent with previous research, WTC workers with probable PTSD were far more likely to report psychological symptoms and behavioral problems in their children compared with WTC workers without probable PTSD. Similar results were observed in victims of the Chornobyl reactor disaster (Bromet et al. 2002).

Unlike most previous findings in civilian trauma survivors of mass disasters or individual traumatic events, the association between post-disaster PTSD and sex was not significant in the present study. Similar findings have been reported in samples of 655 urban police officers (21% female) and 207 exposed disaster workers (11.5% female) as well as in military populations (Sutker et al. 1995). Pole et al. (2001) suggested that selection and/or training factors may help to stress-inoculate women involved in police and military work.

The present study has a number of strengths, including use of standardized assessment instruments, comprehensive psychosocial and medical evaluation, inclusion of both males and females, ethnic diversity of subjects, and large cohort size. In an empirical review of the scientific literature from 1981 to 2001 on disaster victims, Norris et al. (2002) noted problems with small sample sizes as well as demographic and ethnic homogeneity. During that 20-year period, the median sample size for studies related to psychosocial adjustment after disasters was only 159 subjects.

Study limitations include the use of self-administered rather than clinician-administered questionnaires, variability in time to presentation, potential inaccuracy of recall with the passage of time, possible under-reporting of psychological symptoms due to stigma, and lack of assessment within the first few months after 9/11. Because the earliest assessments occurred at least 10 months after the attacks, it is not possible to differentiate delayed onset versus chronic PTSD, and it is not possible to accurately determine rates of acute PTSD. Our self-selected cohort is also a limitation in that we do not know whether workers with psychological symptomatology were more or less likely to enroll. Also, degree of psychological symptomatology may be related to the presence of physical symptoms, and it may be that those with physical illnesses were more likely to seek medical monitoring. Our ability to generalize from this self-selected cohort is enhanced to some extent by its large size, but generalizability is hampered without knowing the true number of WTC rescue and recovery workers nor their sex, race, or ethnicity. Our estimate of 40,000 at-risk workers is considerably lower than the nearly 92,000 estimated by the World Trade Center Registry (Murphy et al. 2007), largely because of the registry’s less stringent criteria for eligibility into their recovery worker cohort. The registry requires a worker or volunteer to have spent one shift at a WTC site between 11 September 2001 and 30 June 2002. The true number of workers and volunteers undoubtedly falls between the two estimates.

Keeping such limitations in mind, the prevalence of probable PTSD among workers in the present study is comparable to that seen in airline-crash recovery workers 13 months after the event (Fullerton et al. 2004) and that observed by North et al. (2002) in firefighters interviewed 34 months after the Oklahoma City bombing.

The present study has a number of implications for public health. Persistent post-disaster mental illness from 10 months to 5 years after the disaster in this cohort underscores the need for long-term mental health screening and treatment programs targeting this population. Chronic mental health disorders constitute a major public health concern. In this cohort, alcohol problems and impairment in occupational, social, and family life were strongly associated with diagnoses of probable PTSD, depression, or panic disorder alone and even with a large number of workers who did not meet criteria for a probable psychiatric disorder but who nevertheless experienced trauma-related psychological symptoms. It is particularly important to screen for comorbid conditions because in our population comorbid conditions were common, and those with these comorbid conditions were at far greater risk for alcohol problems and social dysfunction. The presence of comorbidity may also affect long-term outcome and response to treatment (McFarlane 2002).

Psychiatric disability has effects far beyond the personal suffering of the individual and his or her immediate family. For example, PTSD has substantial economic costs from workdays lost and suboptimal performance. It is estimated that PTSD is associated with approximately 3.6 days of work impairment per month, on average (Breslau et al. 1998). Further, the National Comorbidity Study found that PTSD was associated with marital instability and increased unemployment (Kessler 2000). Taken together, our findings indicate that a substantial public mental health burden exists in the responder population, which puts them at risk for a variety of adverse health and social consequences.

Unfortunately, once psychopathology, such as PTSD, becomes chronic in nature, it can be difficult to treat. The military, recognizing the high frequency of posttraumatic stress symptoms in response to dangerous and life-threatening situations, as well as the costly effects of these symptoms on psychological well being and performance, has recently instituted periodic behavioral health evaluations on all troops returning from Iraq and Afghanistan. Stigma is reduced by requiring every returning soldier to participate in these evaluations. Similarly, rescue and recovery workers after future environmental disasters would likely benefit from routine behavioral health evaluations that are fully integrated into medical evaluations, as well as early treatment when appropriate. Although such efforts should help reduce chronicity of the mental health sequelae of disaster exposure, long-term provision of accessible mental health services for rescue and recovery workers likely should still constitute part of future disaster planning.

Finally, it will be essential in future environmental disasters to understand that mental health problems will almost certainly accompany effects of toxic exposures on physical health. It is also essential that accurate records be kept of the rescue and recovery cohorts so that postdisaster outreach efforts can be improved and better estimates of injury, illness, and disability can be made. Additional rigorous research is needed to better understand and modify the impacts on health of the physical and psychological risk factors that associated with work after environmental disasters (North 2004).

## Figures and Tables

**Table 1:** Prevalence and associations between probable PTSD, major depression, and panic disorder by respondent characteristics.

Characteristic	No.	PTSD (%)	Depression (%)	Panic (%)
Total	10,132	11.1	8.8	5.0
Sex (*p*-value)		NS	< 0.001	< 0.001
Male	8,847	10.9	8.0	4.6
Female	1,285	12.1	14.2	7.6
Age (*p*-value)		< 0.001	< 0.001	NS
≤35 years	2,474	10.2	7.1	4.9
36–45 years	4,085	9.9	7.6	4.7
45–55 years	2,726	13.0	11.5	5.7
≥55 years	847	13.1	11.1	4.4
Marital status (*p*-value)		< 0.001	< 0.001	< 0.001
Single	1,689	11.6	8.5	6.0
Married/partnered	6,709	10.2	7.7	4.3
Separated/divorced	1,277	14.6	14.3	7.2
Widowed	77	13.0	15.6	2.6
Education (*p*-value)		< 0.001	< 0.001	< 0.001
< High school	885	17.2	15.7	8.2
High school	2,565	12.2	10.5	4.8
Attended college	3,785	9.6	6.9	4.2
Graduated college	1,405	8.8	6.0	5.1
Graduate school	786	10.7	7.0	5.6
Race/ethnicity (*p*-value)		< 0.01	< 0.001	< 0.05
White	6,480	10.5	7.9	5.0
Black	1,129	10.8	6.8	3.9
Hispanic	2,230	13.1	12.8	5.8
Asian	131	11.5	6.1	1.5
Other	162	8.0	9.3	4.9
Union member (*p*-value)		< 0.001	< 0.001	< 0.01
Member	8,663	10.5	8.1	4.8
Not a member	1,421	14.6	13.3	6.4
Days at site (*p*-value)		< 0.001	< 0.001	NS
≤ 2 weeks	1,938	9.1	8.2	4.9
Up to 1.5 months	1,976	9.1	6.6	4.7
Up to 3 months	2,170	11.5	9.4	5.3
Up to 5.5 months	1,948	11.9	9.4	4.9
> 5.5 months	1,991	13.3	10.6	5.3
Present 9/11–9/12 (*p*-value)		< 0.001	< 0.001	< 0.001
Yes	6,146	10.2	6.3	4.5
No	3,986	12.5	12.8	5.8

NS, not significant.

**Table 2:** Psychiatric comorbidity and risk for alcohol abuse or emotional disruption of function.

		Alcohol problem		Emotional disruption of function	
Psychiatric condition	No. (%)	No.	OR (CI)^a^	No.	OR (CI)^a^
No PTSD, panic, or depression	8,377 (82.7)	1,264	1.0	406	1.0
Any PTSD, panic, or depression	1,755 (17.3)	498	2.3 (2.0–2.5)	821	17.3 (15.1–19.8)
Probable PTSD only	563 (6.3)	180	2.6 (2.2–3.2)	154	7.4 (6.0–9.1)
PTSD plus either panic or depression	416 (4.7)	126	2.5 (2.0–3.0)	278	39.6 (31.5–50.0)
PTSD plus both panic and depression	145 (1.7)	53	3.2 (2.3–4.6)	118	85.8 (55.8–131.9)
Depression only	368 (4.2)	80	1.6 (1.2–2.0)	187	20.3 (16.2–25.5)
Panic disorder only	199 (2.3)	47	1.7 (1.3–2.4)	40	4.9 (3.4–7.1)
Panic and depression only	64 (0.8)	12	1.3 (0.7–2.4)	44	43.2 (25.2–74.0)
Total	10,132 (100)	1,762		1,227	

a ORs are the odds for reporting an alcohol problem (CAGE scale) or emotional disruption (Sheehan scale) in those with the conditions listed compared to those with no PTSD, panic, or depression (reference group).

**Table 3:** Responder reports of child symptoms during WTC work period and in month before examination in those with and without probable PTSD.

	While on site [no. (%)]			Month before visit [no. (%)]		
Child symptom	No PTSD	PTSD	OR (95% CI)	No PTSD	PTSD	OR (95% CI)
More fearful	2,126 (52.1)	329 (70.4)	2.2 (1.8–2.7)	730 (20.0)	162 (39.0)	2.6 (2.1–3.2)
More clingy	1,509 (37.9)	261 (60.4)	2.5 (2.0–3.1)	773 (21.7)	178 (45.1)	3.0 (2.4–3.7)
More withdrawn	256 (6.5)	110 (25.3)	4.9 (3.8–6.3)	266 (7.5)	76 (19.10)	2.9 (2.2–3.8)
More aggressive	260 (6.5)	121 (27.8)	5.5 (4.3–7.0)	333 (9.4)	96 (24.4)	3.1 (2.4–4.0)
Trouble with sleep	608 (15.2)	158 (36.2)	3.2 (2.6–3.9)	360 (10.1)	102 (26.0)	3.1 (2.4–4.0)
Frequent nightmares	355 (9.0)	140 (32.5)	4.9 (3.9–6.2)	270 (7.6)	80 (20.9)	3.2 (2.4–4.2)
Physical complaints	135 (3.4)	68 (15.7)	5.3 (3.9–7.3)	250 (7.0)	60 (15.4)	2.4 (1.8–3.2)
Change in appetite	202 (5.1)	97 (22.4)	5.4 (4.1–7.0)	276 (7.7)	79 (20.1)	3.0 (2.3–3.9)
Immature behaviors	197 (5.0)	92 (21.4)	5.2 (4.0–6.8)	279 (7.9)	79 (20.3)	3.0 (2.3–3.9)
School behavior problems	267 (6.7)	95 (21.6)	3.8 (3.0–5.0)	309 (8.7)	68 (17.3)	2.2 (1.7–2.9)
Home behavior problems	313 (7.9)	125 (28.3)	4.6 (3.7–5.9)	380 (10.7)	115 (29.1)	3.4 (2.7–4.4)
Poor grades in school	284 (7.2)	98 (22.5)	3.8 (2.9–4.8)	316 (8.9)	68 (17.4)	2.2 (1.6–2.9)

ORs (95% CIs) represent the comparison of those with probable PTSD compared to those who responded and did not have a probable PTSD diagnosis.
